# Killer Cell Immunoglobulin-Like Receptor Haplotype B Modulates Susceptibility to EBV-Associated Classic Hodgkin Lymphoma

**DOI:** 10.3389/fimmu.2022.829943

**Published:** 2022-01-27

**Authors:** Peijia Jiang, Ilja M. Nolte, Bouke G. Hepkema, Marijke Stulp, Anke van den Berg, Arjan Diepstra

**Affiliations:** ^1^ Department of Pathology and Medical Biology, University Medical Center Groningen, University of Groningen, Groningen, Netherlands; ^2^ Department of Epidemiology, University Medical Center Groningen, University of Groningen, Groningen, Netherlands; ^3^ Department of Laboratory Medicine, University Medical Center Groningen, University of Groningen, Groningen, Netherlands

**Keywords:** KIR, HLA class I, susceptibility, EBV, NK cells, CHL

## Abstract

Tumor cells of classic Hodgkin lymphoma (cHL) are derived from antigen presenting B cells that are infected by Epstein Barr virus (EBV) in ~30% of patients. Polymorphic Killer cell immunoglobulin-like receptors (KIRs) expressed on NK cells interact with human leukocyte antigen (HLA) class I and play a key role in immune surveillance against virally infected cells and tumor cells. We investigated the effect of KIR types on cHL susceptibility overall (n=211) and in EBV-stratified subgroups using the Dutch GoNL cohort as controls (n=498). The frequency of the KIR haplotype B subgroup was significantly different between EBV+ and EBV− cHL patients (62% vs. 77%, p=0.04) and this difference was more pronounced in nodular sclerosis (NS) cHL (49% vs. 79%, p=0.0003). The frequency of KIR haplotype B subgroup was significantly lower in EBV+ NS cHL compared to controls (49% vs. 67%, p=0.01). Analyses of known KIR – HLA interaction pairs revealed lower carrier frequencies of *KIR2DS2* – HLA-C1 (29% vs. 46%, p=0.03) and *KIR2DL2* – HLA-C1 (29% vs. 45%, p=0.04) in EBV+ NS cHL patients compared to controls. Carriers of the KIR haplotype B subgroup are less likely to develop EBV+ NS cHL, probably because of a more efficient control over EBV-infected B cells.

## Introduction

Hodgkin lymphoma (HL) is a B-cell derived malignancy most commonly affecting children and young adolescents. Two main subtypes are recognized, i.e. classic HL (cHL) and nodular lymphocyte predominant HL (NLPHL), of which cHL comprises about 95% of all cases. A characteristic feature of cHL is the low percentage of neoplastic cells, i.e. Hodgkin-Reed Sternberg (HRS) cells, surrounded by an abundant infiltrate of reactive immune cells (1). cHL is further classified into four subtypes, i.e. nodular sclerosis (NS), mixed cellularity (MC), lymphocyte rich (LR) and lymphocyte depleted (LD). NS cHL is the most common subtype in high-income countries. In the western world, about 30% of the cHL cases are associated with Epstein-Barr virus (EBV) (2). In these cases, monoclonal EBV genomes are present in all tumor cells suggesting that infection with EBV is an early event. The age-incidence of cHL shows a bimodal pattern with peaks in young adults and individuals aged > 45 (3). EBV+ cases are observed more frequently in children, elderly, male and MC subtype patients (3–5).

HRS cells originate from germinal center B cells and cHL presents in lymph nodes which is an immune cell rich microenvironment (6). The precursor cells need to escape from anti-tumor immune responses, especially those that involve HLA class I and CD8+ cytotoxic T lymphocytes (CTLs) and natural killer (NK) cells. CTLs may recognize tumor specific antigens presented by HLA class I molecules on HRS cells through T cell receptors (TCRs) (7). In general, NK cells recognize presence of HLA class I irrespective of the antigenic peptide and kill HLA class I negative cells (8). Further proof for a critical role of HLA in cHL was obtained by HLA typing and genome-wide association studies (GWAS) that revealed strong associations between cHL and specific HLA alleles and single nucleotide polymorphisms (SNPs) in the HLA region, respectively (9–12). Part of these associations were linked to cHL overall, while others were restricted to the EBV+ cHL subgroup. The most pronounced associations were observed for HLA-A and EBV+ cHL, with HLA-A*01 being a risk allele and HLA-A*02 being a protective allele. These associations are in line with the previously reported ability of HLA-A*02 to induce anti-EBV-specific CTL responses, while these are lacking for HLA-A*01 (13, 14).

**Figure 1:**
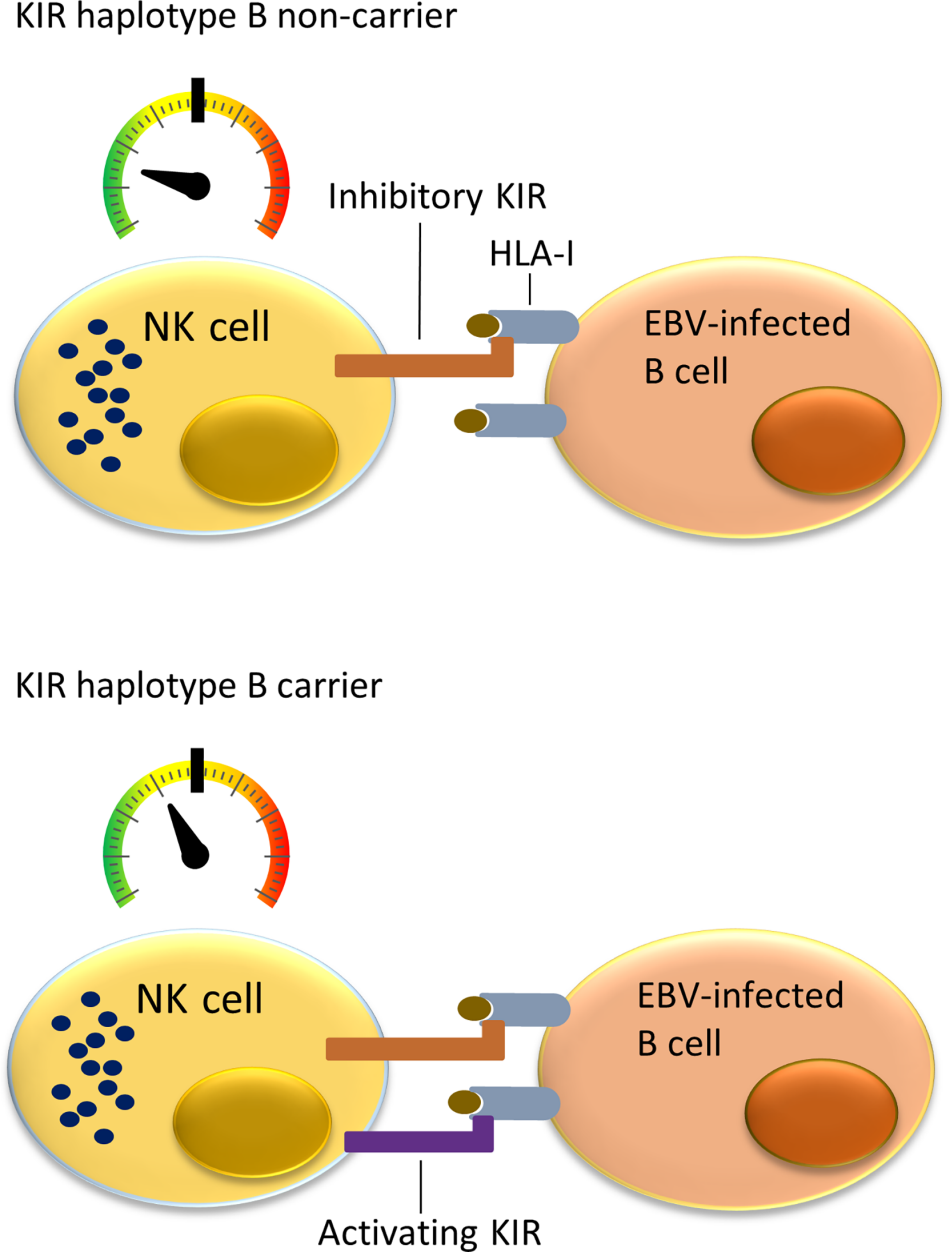
Immune responses to EBV-infected B cells mediated by NK cells and cytotoxic T lymphocytes (CTLs) in KIR haplotype B carriers or non-carriers. NK cells in individuals not carrying KIR haplotype B often do not express a functional activating KIR receptor. The only activating KIR gene which is then present is *KIR2DS4*, which product ligates to a few uncommon HLA class I types and often has an allele preventing translation to protein. This means that NK cells in KIR haplotype B non-carriers have a relatively low level of stimulation (upper panel). In comparison, the multiple activating KIRs in KIR haplotype B carriers provide additional activating signals when binding to HLA class I ligand and stimulate NK cells closer to becoming fully activated and cytotoxic against virally infected cells (lower panel).

NK cells can monitor HLA class I expression on target cells through multiple NK-cell receptors such as killer cell immunoglobulin-like receptors (KIRs). The KIR gene family is highly polymorphic with respect to the sequence and the number of genes, including nine inhibitory (*KIR2DL1*, *KIR2DL2*, *KIR2DL3*, *KIR2DL4*, *KIR2DL5A*, *KIR2DL5B*, *KIR3DL1*, *KIR3DL2*, *KIR3DL3*) and six activating (*KIR2DS1*, *KIR2DS2*, *KIR2DS3*, *KIR2DS4*, *KIR2DS5*, *KIR3DS1*) genes. KIRs can inhibit or activate NK cells dependent on binding to specific ligands, which include amongst others specific HLA class I molecules or epitope subgroups (15–17). The HLA-C types can be divided into two epitope groups, i.e. HLA-C1 and HLA-C2, based on presence of an asparagine (HLA-C1) or lysine (HLA-C2) at position 80 of the α1 domain (18). Well-established receptor – ligand interactions are limited to *KIR2DL1* and *KIR2DS1* interacting with the HLA-C2 epitope (present on C*02, C*04, C*05, C*06, C*15, C*17 and C*18), *KIR2DL2, KIR2DL3*, and *KIR2DS2* recognizing the HLA-C1 epitope (present on C*01, C*03, C*07, C*08, C*12, C*14 and C*16), *KIR3DL1* and *KIR3DS1* recognizing the HLA-Bw4 epitope (present on B*13, B*27, B*37, B*38, B*44, B*49, B*51, B*52, B*57, A*23, A*24, A*25 and A*32), and *KIR3DL2* recognizing both HLA-A*03 and HLA-A*11. The KIR gene region can be broadly divided into two haplotype subgroups. The haplotype A subgroup includes all haplotypes with only one activating KIR gene *KIR2DS4*, which is often not functional, and up to six inhibiting KIR genes. The haplotype B subgroup includes a variable number of inhibiting KIR genes and multiple functional activating genes (19). Each NK cell can express different subsets of the KIR genes resulting in a highly heterogeneous mix of NK cells within each individual (20). Multiple KIR haplotype B-specific activating genes are known to play a protective role in diseases caused by viral infection by the human immunodeficiency virus (HIV), Human Cytomegalovirus (HCMV), hepatitis C virus (HCV) or EBV (21–26). Thus, NK cells in KIR haplotype B subgroup carriers are more easily activated upon viral infection due to presence of multiple activating KIR genes that for example can recognize virally induced cellular stress by interacting with open conformers of HLA-F (21). Previous studies showed a role of NK cells in controlling primary EBV infection (25, 26), and demonstrated a more effective response against EBV infected cells in KIR haplotype B carriers (**Figure 1**). A few studies have investigated associations of KIR genes with cHL but little attention has been paid to KIR associations in EBV stratified cHL subgroups (27–29).

In this study, we hypothesize that KIR haplotype B carriers have a better control over EBV+ B cells resulting in a lower risk of developing EBV+ cHL.

## Materials and Methods

### Patient and Control Samples

A total of 210 cHL patients from the Northern part of the Netherlands were included in this study. We included all available EBV+ cHL patients to maximize power (n = 85, 40%). The remaining 125 cases, including 94 EBV− and 31 cHL cases for which the EBV status was unknown, were selected based on availability of sufficient DNA. Data of the original cohort on sex, age, histological subtype, HLA type, and EBV status have been published in previous studies (11, 30). cHL diagnoses were revised according to the 2017 classification of the World Health Organization by an experienced hematopathologist (31). EBV status was determined by EBV-encoded small RNA (EBER) *in-situ* hybridization using standard protocols.

SNP genotype data of Dutch controls were retrieved from The Genome of the Netherlands (GoNL) dataset (32). KIR types imputed based on whole genome sequencing data for controls were obtained from a previous study (33). We only included the 498 parents and not the children to avoid bias in the allele frequency estimations.

### KIR Typing

KIR typing was done using a commercially available KIR typing kit (Immucor Inc., Norcross, Georgia, United States) on the Luminex 200 platform. For each reaction, 50 ng genomic DNA was mixed with Master Mix in a 96-well plate. Genomic DNA was amplified by PCR and hybridized to the probes provided by the Lifecodes KIR Genotyping kit according to the manufacturer’s protocol. KIR typing calls were assessed using MATCH IT! DNA 1.2.4 (Immucor Inc., Norcross, Georgia, United States). Phenotype (positive or negative) of 12 KIR genes, i.e. *KIR2DL1, KIR2DL2, KIR2DL3, KIR2DL4, KIR2DL5, KIR3DL1, KIR2DS1, KIR2DS2, KIR2DS3, KIR2DS4* (plus two additional isoforms, one with full length, the other with a 22 bp frame-shift deletion), *KIR2DS5* and *KIR3DS1*, were identified for each patient. Patients were stratified into KIR haplotype B subgroup carriers and non-carriers, based on presence or absence of haplotype B-specific KIR genes (*KIR2DL2, KIR2DL5, KIR2DS1, KIR2DS2, KIR2DS3, KIR2DS5*, and *KIR3DS1*).

### HLA Imputation

HLA class I types of GoNL controls were determined using the SNP-based HLA imputation R-package HIBAG (34). The imputed HLA alleles with prediction probability less than 0.8 were excluded. Patients and controls were stratified into three HLA type subgroups based on carriership of specific epitope groups of HLA alleles, i.e. HLA-Bw4+ (including HLA-B*13, B*27, B*37, B*38, B*44, B*49, B*51, B*52, B*57, A*23, A*24, A*25 and A*32), HLA-C1+ (including HLA-C*01, C*03, C*07, C*08, C*12, C*14 and C*16), and HLA-C2+ (including HLA-C*02, C*04, C*05, C*06, C*15, C*17 and C*18).

### Statistical Analysis

Chi square tests were used for association analyses of KIR gene or haplotype carriership in cHL overall or EBV stratified patient groups and for KIR receptor – HLA ligand interaction analysis. A p-value < 0.05 was regarded as significant.

## Results

### Characteristics of Patients

The median age of controls was 62 years (range: 43 to 87) and for cHL patients it was 32 years (range: 14 to 89) (**Table 1**). Consistent with previous studies, there were more males than females (76% vs. 24%) in the EBV+ cHL subgroup (4, 35). The most common subtype in both cHL overall and EBV-stratified subgroups was the NS subtype.

**Table 1:** Characteristics of controls and patients.

Characteristic		GoNL controls (n=498)	cHL Patients			
			Total (n=210)	EBV+ (n=85)	EBV− (n=94)	EBV Unknown (n=31)
Age at diagnosis, median (range)		62 (43–87)	32 (14-89)	35 (14-70)	32 (15-89)	31 (17-61)
Sex, no. (%)						
	male	250 (50%)	123 (58%)	65 (76%)	46 (49%)	12 (39%)
	female	248 (50%)	87 (41%)	20 (24%)	48 (51%)	19 (61%)
Subtype, no. (%)						
	NS		153 (73%)	49 (58%)	82 (87%)	22 (71%)
	LR		4 (2%)	3 (4%)	1 (1%)	0 (0%)
	MC		25 (12%)	22 (26%)	1 (1%)	2 (6%)
	LD		0 (0%)	0 (0%)	0 (0%)	0 (0%)
	NOS		28 (13%)	11 (13%)	10 (11%)	7 (23%)

NS, nodular sclerosis; LR, lymphocyte-rich; MC, mixed cellularity; LD, lymphocyte depleted; NOS, not otherwise specified; GoNL, genome of the Netherlands.

### KIR Typing

KIR haplotype B subgroup carriers accounted for 72% of the total cHL cohort and carrier frequencies of B-specific genes i.e. *KIR2DS2*, *KIR2DL2*, *KIR2DL5*, *KIR2DS3*, *KIR2DS5*, *KIR3DS1* and *KIR2DS1*, ranged from 26% to 54% (**Supplementary Table 1**). Carrier frequencies of the other KIR genes were more than 90%. The two alleles of *KIR2DS4*, *2DS4WT* (wild type) and *2DS4DEL* (resulting in a stop codon), were present in 47% and 73% of the patients, respectively. No significant differences of *KIR* gene frequencies were observed between cHL patients overall and Dutch GoNL controls (**Supplementary Table 1**).

### Differences Between EBV+ and EBV− cHL

**Figure 2:**
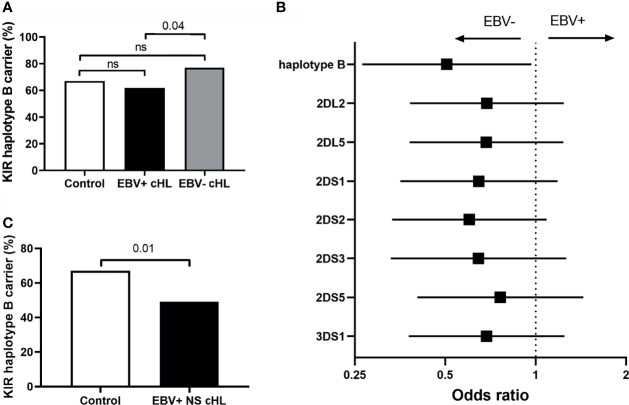
Association between KIR haplotype B and EBV status in cHL. **(A)** Bar plot showing the KIR haplotype B frequencies in controls (white), EBV+ (black) and EBV− (grey) cHL subgroups, ns: not significant. **(B)** The odds ratios (black squares) and 95% confidence intervals (whiskers) of KIR haplotype B and haplotype B-specific genes to have EBV− or EBV+ cHL are shown in the forest plot. A higher odds ratio means a higher risk for EBV+ cHL. **(C)** Bar plot showing the KIR haplotype B frequencies in controls (white) and EBV+ nodular sclerosis (NS) (black) cHL subgroups.

To test our hypothesis, we compared the frequency of haplotype B subgroup carriers between EBV-stratified cHL subgroups and the GoNL controls. Although no significant differences were observed between EBV stratified groups and GoNL controls, we did find a significantly lower haplotype B frequency in EBV+ compared to EBV− cHL (62% vs. 77%, p = 0.04) (**Figure 2A**). The odds ratio (OR) of having EBV+ cHL in haplotype B carriers was 0.51 compared to having EBV− cHL. To determine if the difference was caused by a single haplotype B-subgroup specific gene, we also analyzed the frequencies of individual haplotype B-subgroup specific genes in EBV-stratified subgroups. All seven haplotype B-subgroup specific genes showed a similar pattern with lower carrier frequencies in EBV+ than EBV− cHL, albeit not significant (**Figure 2B**). The odds ratios were similar, with the lowest OR for *KIR2DS2* (0.60) and the highest OR for *KIR2DS5* (0.76) (**Figure 2B**).

As the EBV status of cHL is associated with subtype, age and sex of the cHL patients, we next analyzed KIR haplotype B subgroup frequencies in age, sex, and histological subtype stratified subgroups. The difference in KIR haplotype B subgroup carrier frequency was most pronounced in NS cHL (49% vs. 79%, p = 0.0003). Non-NS cHL showed a pattern opposite to the NS subgroup, albeit non-significant. The KIR haplotype B subgroup carrier frequencies were also significantly different in females (p = 0.01) and cHL patients younger than 45 (p = 0.03), while no differences were observed in males and cHL patients of 45 and older, respectively (**Table 2**, **Supplementary Figures 1A–C**). Analysis of individual KIR haplotype B-subgroup specific genes revealed similar patterns in the NS subgroup and again opposite patterns in the non-NS subgroup (**Supplementary Figure 1D**). Specifically testing EBV+ NS versus GoNL controls revealed that the KIR haplotype B subgroup protects against the development of this cHL subgroup (49% vs. 67%, p = 0.01) (**Figure 2C**).

**Table 2:** Frequency differences of KIR haplotype B carriers between EBV+ and EBV− cHL in subtype-, sex- and age-stratified subgroups.

cHL subgroup		EBV+		EBV−		*P* value (EBV+ vs. EBV−)
		N	KIR B Carriers (%)	N	KIR B Carriers (%)	
Subtype						
	NS	49	24 (49%)	82	65 (79%)	0.0003
	Non-NS	36	29 (81%)	12	7 (58%)	ns
Sex						
	male	65	44 (68%)	45	35 (76%)	ns
	female	20	9 (45%)	48	37 (77%)	0.01
Age						
	Age<45	61	39 (64%)	73	59 (81%)	0.03
	Age>=45	24	14 (58%)	21	13 (62%)	ns

NS, nodular sclerosis; ns, not significant.

### KIR–HLA Interaction

HLA imputation of 498 GoNL controls resulted in 480, 456 and 487 well-imputed individuals for HLA-A, HLA-B and HLA-C, respectively. Allele frequencies of HLA-A and HLA-B for well-imputed controls were similar with direct typing data from our previous study (**Supplementary Table 2**) (11). No significant differences were found in allele frequencies of known KIR – HLA pairs between patients and controls (**Supplementary Table 3**).

Based on the differences observed for the KIR haplotype B associations especially within EBV+ NS cHL, we also studied associations of known KIR – HLA pairs in this subgroup. This revealed significant associations for *KIR2DL2* and HLA-C1 (29% vs. 45%, p = 0.04) and for *KIR2DS2* and HLA-C1 (**Table 3**).

**Table 3:** KIR receptor – HLA ligand interactions in EBV+ NS cHL patients and GoNL controls.

KIR	HLA	Patients N (%)	GoNL Controls N (%)	p value
*KIR2DL2*+	HLA-C1+	14 (29%)	217 (45%)	**0.04**
*KIR2DL3*+	HLA-C1+	42 (88%)	387 (80%)	0.20
*KIR3DL1*+	HLA-Bw4+	29 (59%)	299 (65%)	0.40
*KIR2DS1*+	HLA-C2+	6 (13%)	98 (20%)	0.20
*KIR2DS2*+	HLA-C1+	14 (29%)	223 (46%)	**0.03**
*KIR3DS1*+	HLA-Bw4+	7 (14%)	118 (26%)	0.08

Significant differences with p < 0.05 are shown as bold. GoNL, genome of the Netherlands.

## Discussion

In this study, we explored associations between KIR and cHL in EBV-stratified subgroups and the interaction of specific KIR genes with their known HLA ligands. We found that KIR haplotype B subgroup protects against the development of EBV+ NS cHL. In addition, *KIR2DL2* – HLA-C1 and *KIR2DS2* – HLA-C1 were observed at significantly lower carrier frequencies in EBV+ NS cHL patients compared to controls. These two KIR genes belong to the KIR B haplotype subgroup, which protects against EBV+ NS cHL.

Infection of B cells with EBV is considered to be an early event in the development of EBV+ cHL based on presence of clonal EBV genomes in HRS cells (36). In healthy individuals, the number of EBV infected cells is well-controlled by the immune system to a rate of 1-50 infected B cells per million B cells in the peripheral blood (37). Previous studies showed that elevated levels of antibodies against EBV capsid antigen and early antigen D were increased around 4 years before EBV+ cHL diagnosis (38). Moreover, the risk of developing EBV+ cHL was increased in individuals suffering from infectious mononucleosis (IM) (39, 40). Together this indicates that an increase of EBV infected B cells may increase the risk of EBV+ cHL. NK cells were shown to play an essential role in the control of EBV infected B cells and loss of NK cells increases the incidence of lymphoma in a mouse model (41, 42). Not only NK cells but also KIRs were reported to be involved in EBV-associated diseases such as IM and EBV infection after hematopoietic stem-cell transplantation (25, 26). The frequency of the KIR haplotype B specific gene, *KIR2DS2*, was lower in IM patients than in controls, suggesting a protective role of this KIR haplotype B specific gene (25). These data and our results fit with our hypothesis, proposing that KIR haplotype B subgroup might be associated with a better control of EBV infected B cells during primary infection, resulting in a lower number of latently infected B cells and a lower chance of developing EBV+ cHL.

Although the model can explain susceptibility effects by decreasing the number of EBV+ cHL precursor cells, it does not explain why this effect appears to be specific for the NS subtype. This subtype is characterized by clustering of large HRS cells in nodules and the occurrence of scar-like fibrosis. In general, NK cells are present in the tumor microenvironment of cHL, but at numbers lower than in normal lymph nodes and in non-Hodgkin lymphomas. Moreover, the number of NK cells were reported to be lower in EBV+ cHL cases (7 out 10 cases were NS subtype) compared to EBV− cHL (43). The number of circulating NK cells in cHL patients is low and these NK cells display a decreased cytotoxicity (44). These observations suggest that NK cells are actively suppressed by the HRS cells. One of the mechanisms that HRS cells can use is the production of the immunosuppressive cytokine Transforming Growth Factor-β (TGF-β), which not only inhibits NK cells but also induces the fibrosis characteristic of the NS subtype (45–47). This might indicate that NK cell function in KIR haplotype B carriers who do start to develop EBV+ cHL, is most likely inhibited by mechanisms other than TGF-β, resulting in non-NS subtype. Potential other mechanisms that might lead to NK cell inhibition include production of the immunosuppressive cytokine IL10 and expression of HLA-G. Expression levels of IL10 were indeed reported to be higher in EBV+ than EBV− cHL (48–50). Combined with the lower number of NK cells in EBV+ cHL cases (43), this supports a stronger impairment of NK cells in EBV+ cHL. The MC subtype is the second most common subtype and has no fibrosis, but unfortunately, the number of MC patients in our study was too small to do a meaningful analysis of this subgroup. To further explore this issue, a larger study is needed that not only considers KIR type and histological subtype, but also studies the role of TGF-β, IL-10 and other alternative mechanisms to suppress NK cells.

KIRs are dependent on the interaction with their ligands, which include mainly certain HLA class I alleles. We tested interaction of both the receptor and the ligand of known receptor ligand pairs, as presence of both genes can lead to a functional interaction and subsequent triggering of NK cells to either exhibit inhibitory or activating signals towards killing of the target cells. However, no significant differences were observed between controls and cHL patients overall, consistent with previous studies (27, 28). Nevertheless, we did find significant differences in the carrier frequencies of both *KIR2DL2* and *KIR2DS2* together with HLA-C1 in EBV+ NS cHL cases. So, both the inhibitory and the activating KIR receptor are associated with HLA-C1. Although signaling of inhibitory KIRs is dominant over signaling *via* activating KIRs, the restricted KIR expression pattern, with one to three KIR genes per NK cell, will result in a substantial proportion of NK cells that only have the activating KIR, and these NK cells will be capable of controlling the number of circulating EBV+ B cells. This can lead to lower numbers of EBV-infected B cells and thereby reduce the risk to develop EBV+ NS cHL.

To put our KIR and HLA typing analyses into perspective we looked at previously published studies in HL. In a family study by Besson et al. *KIR2DS1* and *KIR3DS1* were identified as protective factors in 90 cHL cases and 255 first-degree siblings. However, this could not be replicated in their case – control study including 68 patients (29). In a case – control study including 41 cHL cases and 120 controls from a Lebanese population, also no associations of KIR genes with cHL were reported (28). Consistent with these earlier studies, we did not find any association of specific *KIR* genes with cHL overall in our data either. In a third more recent study in 135 cHL cases and 221 controls, the authors showed a protective role for homozygous KIR haplotype A in homozygous HLA-C1 cHL patients (27). In addition, they reported a significant association between presence of *KIR3DS1* or *KIR3DL1* and absence of HLA alleles belonging to the HLA-Bw4 epitope group. In our data, we could only confirm the higher frequency of *KIR3DS1* in cHL patients who did not carry HLA-Bw4 (20.5% vs. 11.6%, p = 0.002). The explanation for this finding is unclear because HLA-Bw4 is known to be a ligand of *KIR3DS1* and its presence instead of absence would be expected to affect susceptibility. Of note, it has been suggested that *KIR3DS1* ligands other than HLA-Bw4 exist (21).

So far, this is the largest study to investigate the association between KIR and cHL, focusing specifically on EBV stratified cases. Although we aimed at including as many EBV+ cases as possible, the finding of a specific association in the NS subtype, reduced our power. This emphasizes the need of larger studies including sufficient number of MC cases to fully elucidate the relevance of *KIR* gene associations. In addition, we cannot exclude a potential bias in *KIR* frequencies caused by the inclusion strategy of the EBV− patient group.

To conclude, EBV+ NS cHL is less common in KIR haplotype B subgroup carriers as compared to non-haplotype B individuals. Our findings suggest that KIR haplotype B subgroup modulates cHL susceptibility in an EBV-dependent way.

## Data Availability Statement

The original contributions presented in the study are included in the article/**Supplementary Material**. Further inquiries can be directed to the corresponding author.

## Ethics Statement

The study was approved by ethical committee of the University Medical Centre Groningen on November 10, 2011 (ethic code METc 2004/219). Written informed consent to participate in this study was provided by the participants’ legal guardian/next of kin.

## Author Contributions

PJ, AB, and AD designed the study and performed data analysis and interpretation. IN did the statistical analyses. MS and BH contributed to KIR and HLA typing. PJ did HLA imputation analysis. PJ wrote the manuscript. The study was performed under the supervision of AB and AD. All authors have read and agreed to the published version of manuscript.

## Funding

This work was supported by a grant from the Dutch Cancer Society (KWF RUG 2014-6698).

## Conflict of Interest

The authors declare that the research was conducted in the absence of any commercial or financial relationships that could be construed as a potential conflict of interest.

## Publisher’s Note

All claims expressed in this article are solely those of the authors and do not necessarily represent those of their affiliated organizations, or those of the publisher, the editors and the reviewers. Any product that may be evaluated in this article, or claim that may be made by its manufacturer, is not guaranteed or endorsed by the publisher.
