# Supplementary material for: Killer Cell Immunoglobulin-Like Receptor Haplotype B Modulates Susceptibility to EBV-Associated Classic Hodgkin Lymphoma
Source: Front Immunol. 2022 Jan 27;13:829943. doi: 10.3389/fimmu.2022.829943 (PMC8828906; doi:10.3389/fimmu.2022.829943)
Supplement: Supplementary file 1 [file DataSheet_1.docx]

**Supplementary Materials**

**Killer cell immunoglobulin-like receptor haplotype B modulates susceptibility to EBV-associated classic Hodgkin lymphoma**

Peijia Jiang, Ilja M. Nolte, Bouke G. Hepkema, Marijke Stulp, Anke van den Berg, Arjan Diepstra

**Supplementary Table 1.** Frequencies of carriers of KIR haplotype B and KIR genes in patients and GoNL controls (1).

| **KIR** | **cHL cases**  **(n=210)** | **GoNL prediction using WGS data ^(1)^ (n=498)** | **p value** |
| --- | --- | --- | --- |
| Haplotype B | 72% | 67% | ns |
| *2DS2* | 54% | 53% | ns |
| *2DL2* | 53% | 51% | ns |
| *2DL3* | 95% | 92% | ns |
| *2DL5* | 55% | 47% | ns |
| *2DS3* | 31% | 27% | ns |
| *2DS5* | 26% | 28% | ns |
| *2DL1* | 99% | 97% | ns |
| *2DL4* | 100% | 100% | ns |
| *3DL1* | 93% | 96% | ns |
| *3DS1* | 44% | 37% | ns |
| *2DS1* | 42% | 35% | ns |
| *2DS4* | 93% | 95% | ns |
| *2DS4WT* | 47% | - |  |
| *2DS4DEL* | 73% | - |  |

Chi-square test were used for statistical analyses. GoNL: Genome of the Netherlands dataset, ns: not significant, WGS: whole genome sequencing, WT: wildtype, DEL: deletion variant of the 2DS4 gene resulting in a non-functional isoform of 2DS4 due to frameshift.

(1). Roe et al. Front Immunol. 2020

**Supplementary Table 2.** Frequencies of HLA-A and HLA-B alleles between HLA imputation and direct HLA typing (2).

| HLA-A allele | Imputation of GoNL controls (n=480) (%) | Direct typing of UMCG controls (n=7554) ^(2)^ (%) |  | HLA-B allele | Imputation of GoNL controls (n=458) (%) | Direct typing of UMCG controls (n=7554) ^(2)^ (%) |
| --- | --- | --- | --- | --- | --- | --- |
| 1 | 19.1 | 18.8 |  | 7 | 14.4 | 16.8 |
| 2 | 30.9 | 34 |  | 8 | 14.4 | 14.2 |
| 3 | 16.3 | 17.8 |  | 13 | 2.4 | 2.2 |
| 11 | 4.7 | 5.2 |  | 14 | 2.3 | 1.5 |
| 23 | 2.1 | 1.1 |  | 15 | 10.4 | 10.1 |
| 24 | 8.2 | 8.9 |  | 18 | 2.8 | 3.2 |
| 25 | 0.7 | 0.6 |  | 27 | 2.8 | 4.3 |
| 26 | 1.6 | 1.8 |  | 35 | 9.4 | 8.8 |
| 29 | 4 | 2.5 |  | 37 | 2.3 | 1.9 |
| 30 | 1.7 | 1.3 |  | 38 | 1.5 | - |
| 31 | 3.1 | 2.9 |  | 39 | 1.6 | 2.1 |
| 32 | 3.3 | 3.5 |  | 40 | 7.9 | 9.3 |
| 33 | 0.5 | - |  | 41 | 0.2 | 0.7 |
| 34 | 0.1 | - |  | 44 | 13.3 | 11.7 |
| 66 | 0.1 | - |  | 45 | 0.4 | - |
| 68 | 3.6 | - |  | 47 | 0.2 | - |
|  |  |  |  | 49 | 0.8 | 0.6 |
|  |  |  |  | 50 | 0.8 | - |
|  |  |  |  | 51 | 5.7 | 5.1 |
|  |  |  |  | 52 | 0.4 | - |
|  |  |  |  | 53 | 0.1 | - |
|  |  |  |  | 55 | 1.7 | - |
|  |  |  |  | 56 | 0.3 | - |
|  |  |  |  | 57 | 3.1 | 2.9 |
|  |  |  |  | 58 | 0.7 | - |

Controls with HLA imputation probability < 0.8 are excluded, GoNL: Genome of the Netherlands dataset.

(2). Huang et al. Plos One 2012

**Supplementary Table 3.** **Frequencies of presence of well-known KIR – HLA receptor-ligand pairs between controls and patients**

| **KIR** | **HLA** | **Patients**  **No. (%)** | **GoNL Controls**  **No. (%)** | **p value** |
| --- | --- | --- | --- | --- |
| *KIR2DL2*+ | HLA-C1+ | 101 (49%) | 217 (45%) | 0.51 |
| *KIR2DL3*+ | HLA-C1+ | 174 (84%) | 387 (80%) | 0.99 |
| *KIR3DL1*+ | HLA-Bw4+ | 116 (55%) | 299 (65%) | 0.27 |
| *KIR2DS1*+ | HLA-C2+ | 43 (21%) | 98 (20%) | 0.31 |
| *KIR2DS2*+ | HLA-C1+ | 102 (49%) | 223 (46%) | 0.55 |
| *KIR3DS1*+ | HLA-Bw4+ | 49 (23%) | 118 (26%) | 0.21 |

Chi-square test were used for statistical analyses. GoNL: Genome of the Netherlands dataset


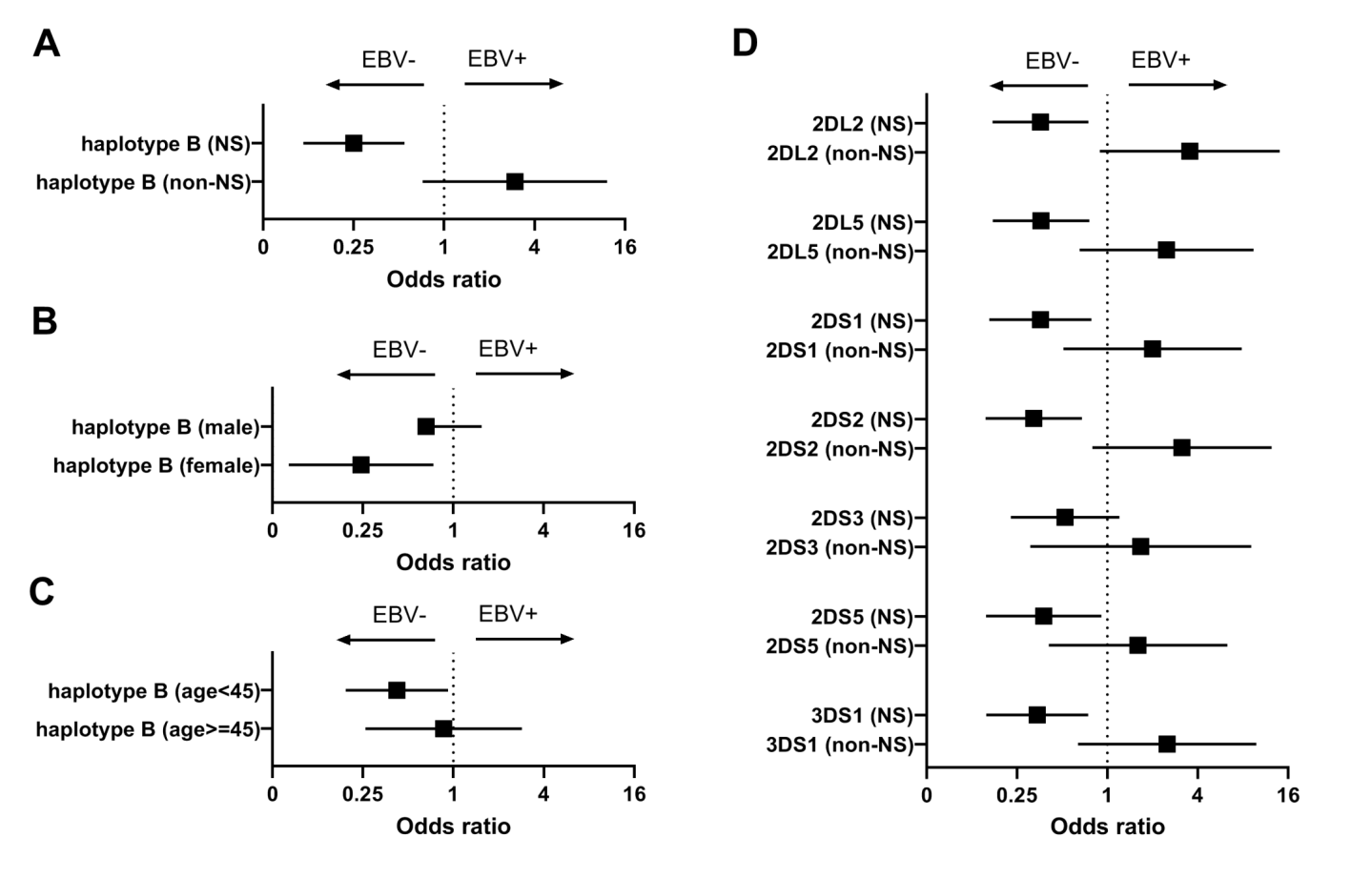
**Supplementary Figure 1. Association of KIR haplotype B and B-specific genes with EBV status in cHL subgroups.** The graphs show differences of KIR haplotype B frequency between EBV+ and EBV- cHL in subtype-stratified (A), sex-stratified (B) and age-stratified (C) cHL subgroups. Different frequencies of KIR haplotype B-specific genes between EBV+ and EBV- subgroups are shown in subtype-stratified patients (D). The black squares reflect the odds ratios and lines represent 95% confidence intervals. NS: nodular sclerosis.

**References**

1. Roe D, Kuang R. Accurate and efficient KIR gene and haplotype inference from genome sequencing reads with novel K-mer signatures. Front Immunol. 2020;11:583013. doi: 10.3389/fimmu.2020.583013.
2. Huang X, Kushekhar K, Nolte I, Kooistra W, Visser L, Bouwman I, et al. HLA associations in classical Hodgkin lymphoma: EBV status matters. PLoS One. 2012;7:e39986. doi: 10.1371/journal.pone.0039986.
